# Subcutaneous Infection with *Dirofilaria* spp. Nematode in Human, France

**DOI:** 10.3201/eid1911.130606

**Published:** 2013-11

**Authors:** Mark L. Eberhard

**Affiliations:** Centers for Disease Control and Prevention, Atlanta, Georgia, USA

**Keywords:** subcutaneous dirofilariasis, Dirofilaria immitis, Dirofilaria repens, Dirofilaria spp., filariasis, nematodes, France, zoonoses, parasites, dirofilariasis, molecular typing, morphology

**To the Editor:** The article by Foissac et al. titled Subcutaneous infection with *Dirofilaria immitis* nematode in human, France (*1*) presents an interesting and challenging diagnostic dilemma. The paper described, but did not illustrate, the worm as having a strongly ridged external surface of the cuticle—a feature known not to exist on *Dirofilaria immitis*, the dog heartworm. However, molecular sequencing of the specimen demonstrated much closer similarity to *D. immitis* than to *D. repens*, the most common cause of zoonotic subcutaneous dirofilariasis infection in Europe.

Well-described morphologic features of parasites, including in tissue sections, have long been the standard for diagnosis. More recently, molecular diagnostics have helped in many of these difficult cases. However, in some cases, the morphology and molecular diagnosis are discordant. On the basis of the data in the article, the worm does not seem to represent *D. repens*. A more likely possibility is some other species for which no sequences are yet available for comparison. In such a worm, the regions sequenced must be similar to *D. immitis*, and distinct from *D. repens*, to achieve the observed results.

When one encounters a case such as this, where well-validated morphologic features (Figure) are contradictory to the molecular analysis, one must exercise caution in arriving at a final diagnosis. One disadvantage of morphologic and molecular diagnostics is an absence of information on poorly described and characterized pathogens or new pathogens that have yet to be identified. No good algorithm exists to resolve these conflicts other than to explore all possibilities. The diagnosis in the described case is probably best left as a *Dirofilaria* species of the *Dirofilaria* (*Nochtiella*) type, members of which exhibit marked cuticular ridging, and not *D.* (*Dirofilaria*) *immitis* type, members of which have as a feature an absence of cuticular ridging.

**Figure:**
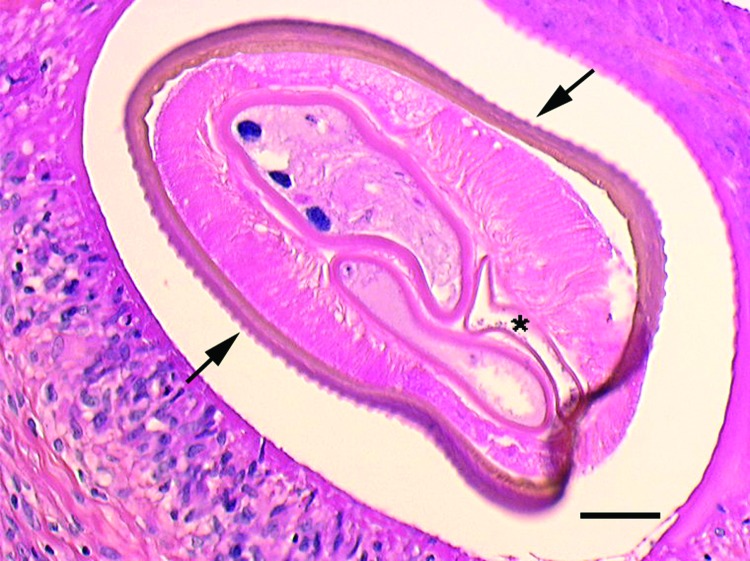
Cross-section of the filarial nematode seen in the subcutaneous nodule on the thigh of a woman in France. The features, as described in the original report (*1*), include prominent, longitudinal ridging of the cuticle (arrows), 2 reproductive tubes, and the intestine (asterisk). Scale bar indicates 50 µm. Image courtesy of Jean-Philippe Dales.
